# Assessing the Predictive Value of Serum Vitamin D Levels for Hip Fracture Risk in Older Adults and Identifying Associated Risk Factors

**DOI:** 10.7759/cureus.82603

**Published:** 2025-04-19

**Authors:** Zahir Khan, Muhammad Arsalan Azmat Swati, Shah Zeb, Ashraf, Rumman, Amir Sohail, Kamran Butt

**Affiliations:** 1 Orthopedic Surgery, Mardan Medical Complex, Medical Teaching Institution (MTI) Mardan, Mardan, PAK; 2 Orthopedic Surgery, Bacha Khan Medical College, Medical Teaching Institution (MTI) Mardan, Mardan, PAK; 3 Orthopedics and Traumatology, Mardan Medical Complex, Medical Teaching Institution (MTI) Mardan, Mardan, PAK; 4 Internal Medicine, Mardan Medical Complex, Medical Teaching Institution (MTI) Mardan, Mardan, PAK; 5 Internal Medicine, Bacha Khan Medical College, Medical Teaching Institution (MTI) Mardan, Mardan, PAK; 6 Research and Development, Pro-Gene Diagnostics and Research Laboratory, Mardan, PAK; 7 Pulmonary Medicine, Mardan Medical Complex (MMC) Medical Teaching Institution (MTI) Mardan, Teaching Hospital, Mardan, PAK; 8 Pharmacovigilance/Active Drug Safety Monitoring and Management, Mardan Medical Complex (MMC) Teaching Hospital, Peshawar, PAK; 9 Orthopedics, Akhtar Saeed Medical and Dental College, Lahore, PAK

**Keywords:** comorbidities, elderly population, hip fractures, supplementation, vitamin d deficiency

## Abstract

Background

As the elderly population continues to grow globally, the incidence of hip fractures among this demographic is becoming an increasingly pressing public health issue. Hip fractures often result from a complex interplay of factors, including sociodemographic variables, clinical comorbidities, medication use, physical activity, and vitamin D levels. Understanding these factors is essential to mitigating the risk of hip fractures in older individuals.

Objective This study aims to explore the relationship between vitamin D levels and hip fracture risk in elderly patients, identifying the clinical, demographic, and environmental factors contributing to fracture risk, with a particular focus on the role of vitamin D in bone health.

Methodology This cross-sectional observational study was conducted at Mardan Medical Complex between January 2024 and February 2025. A total of 119 older adult patients (aged 50 years and above) with confirmed hip fractures were included. Demographic data, comorbidities, vitamin D levels, and relevant biomarkers were collected. Serum 25-hydroxyvitamin D [25(OH)D] levels were measured using electrochemiluminescence immunoassay. Statistical analysis, including ANOVA, Mann-Whitney U tests, and decision tree modeling, was employed to assess the relationship between vitamin D and other variables, including BMI, mobility, supplementation, and comorbidities.

Results The study found a high prevalence of vitamin D deficiency among older adult patients with hip fractures, with the average serum vitamin D level measured at 15.01 ± 5.51 ng/mL, significantly lower than the recommended levels. Key factors such as Body Mass Index (BMI) (28.27 ± 5.67) and mobility status (59 patients, 49.58% using mobility aids), as well as comorbidities like diabetes (76 patients, 63.87%) and chronic kidney disease (18 patients, 15.13%), were significantly associated with lower vitamin D levels. Vitamin D supplementation, with an average intake of 505.99 ± 284.26 IU/day, was taken by 68 patients (57.14%) and showed a positive effect on serum levels, but the variability in response (ranging from non-users to varying doses) highlighted the need for personalized supplementation strategies. Additionally, age (67.53 ± 10.51 years) and gender (69 males, 57.98%, and 50 females, 42.02%) did not show a significant impact on vitamin D levels or fracture risk in this study.

Conclusion This study supports the importance of vitamin D as a modifiable risk factor for hip fractures in older adult patients. It underscores the need for comprehensive public health strategies, including regular vitamin D screening, targeted supplementation, and management of comorbidities, to reduce fracture risk in aging populations.

## Introduction

The global phenomenon of population aging has significant repercussions on health outcomes in the elderly, notably the increase in hip fractures and associated health challenges. By 2050, the World Health Organization (WHO) predicts that the elderly population will exceed 2 billion, more than double the approximately 1 billion recorded in 2020 [1]. A critical public health concern arises as the incidence of hip fractures continues to grow alongside this demographic shift. Hip fractures are linked to considerable morbidity, prolonged hospitalization, and diminished quality of life [2]. Indeed, annual hospitalizations for hip fractures in the United States alone approach 300,000, a concerning figure expected to rise as current demographic trends continue [1].

The underlying factors contributing to hip fracture risk are numerous and multifaceted. Osteoporosis stands as a primary condition characterized by decreased bone mineral density (BMD), which fosters fragility and heightens the likelihood of fractures, even under minor trauma [3,4]. However, the complexity of hip fractures extends beyond osteoporosis; other clinical comorbidities, medication use, physical activity levels, and environmental elements also critically influence fracture risk [5]. For instance, conditions such as diabetes, cardiovascular diseases, and neurological disorders can exacerbate vulnerability to falls and fractures, demonstrating the need for a holistic view of hip fracture prevention [5,6].

Vitamin D deficiency emerges as a central element affecting bone health, significantly influencing the risk of hip fractures in the elderly [7,8]. Vitamin D is critical for calcium regulation and bone health, facilitating the absorption of calcium and maintaining serum calcium levels necessary for optimal bone mineralization [9,10]. Research shows that inadequate vitamin D levels contribute to a spectrum of skeletal ailments, including osteoporosis and osteomalacia, both of which increase fracture risk [11,12]. Additionally, vitamin D's physiological role extends to muscle function, with deficiency correlating with decreased muscle strength and increased fall risks, further compounding fracture risks [13].

The elderly population is particularly susceptible to vitamin D deficiency due to various factors, notably diminished skin synthesis from sunlight, inadequate dietary intake, and changes in renal function that impair conversion of vitamin D to its active form [3,4]. For example, studies indicate that approximately 86% of screened elderly individuals show low levels of 25-hydroxyvitamin D [25(OH)D], with a significant proportion displaying severe deficiency [14]. This situation is compounded by the common use of medications such as corticosteroids and anticonvulsants, which can hinder vitamin D metabolism and exacerbate deficiency [5].

Research also highlights the seasonal variation in vitamin D levels among the elderly population, demonstrating how fluctuations affect bone turnover and subsequently fracture risks [15]. One study reported that lower serum vitamin D levels during winter months coincide with an uptick in fragility fractures, underscoring the need for consistent vitamin D monitoring across different seasons [16]. Interventions to mitigate vitamin D deficiency in this demographic have shown promise; combined supplementation with vitamin D and calcium for extended periods has been associated with substantial reductions in hip fracture incidence among elderly women [17,8].

In addressing the overarching aim of this study, which is to elucidate the relationship between vitamin D levels and hip fracture risk, it is imperative to consider the interaction of various risk factors. Clinical comorbidities such as diabetes and neurological impairments appear to compound the effects of vitamin D insufficiency on bone health, indicating a need for multifactorial intervention strategies [5,13]. Additionally, maintaining adequate levels of vitamin D in the elderly could enhance not only bone health but also overall physical function, potentially reducing the incidence of falls that lead to fractures [18,3].

Public health strategies must engage in preventive measures that advocate for widespread vitamin D screening and supplementation in at-risk elderly populations. Education regarding dietary sources of vitamin D and the importance of sun exposure remains paramount in fostering better health outcomes for this demographic [19,8]. As the aging population continues to burgeon, it becomes increasingly critical that healthcare systems adapt to address the complex interplay of factors contributing to hip fracture risks, with a focus on promoting optimal vitamin D levels as a foundation for improving bone health in older adults [11].

Ultimately, understanding and addressing the multifactorial nature of hip fractures will not only improve individual patient outcomes but will also alleviate the broader strain on healthcare resources as the population ages. Tools such as comprehensive health assessments that account for vitamin D levels alongside other risk factors are essential in formulating effective interventions for fracture prevention. This study encourages a reconceptualization of public health approaches to aging, emphasizing the critical role of vitamin D in skeletal health, thereby positioning it as a vital component in the preventive arsenal against hip fractures in the elderly [20,12].

The intersection of aging, vitamin D deficiency, and hip fracture risk presents a significant public health challenge that requires a multifaceted approach for management and prevention. As the global demographic landscape shifts, proactive measures to enhance vitamin D status among the elderly must be prioritized to combat the rising incidence of hip fractures and improve the quality of life for aging populations around the world.

## Materials and methods

Study design and setting

This cross-sectional observational study was conducted at the Department of Orthopedics, Mardan Medical Complex, from January 2024 to February 2025. The study aimed to explore the role of vitamin D levels as a potential predictor of hip fracture risk in elderly patients. A convenience sampling technique was employed to recruit participants who presented to the hospital for surgery due to hip fractures during the study period.

Inclusion and exclusion criteria

The study population comprised older adults aged 50 years and above who were admitted with a confirmed hip fracture and scheduled for surgical management during the study period. Inclusion required that participants have a documented serum 25(OH)D level obtained within the first 72 hours of hospitalization, along with accessible baseline laboratory data, including hematological and biochemical profiles, to enable thorough evaluation of potential confounding variables. Eligible individuals were also required to be clinically stable, capable of providing written informed consent, and able to participate in structured interviews and assessments. Patients were excluded if they had underlying conditions known to interfere with vitamin D metabolism, such as recent exposure to immunosuppressive agents, systemic corticosteroid therapy within the prior three months, or recent major surgery. Those who had received high-dose vitamin D supplementation (>4000 IU/day) were also excluded to avoid skewed serum 25(OH)D measurements. Additional exclusion criteria included participation in other clinical trials involving bone health or investigational therapies, as well as incomplete medical documentation or absence of timely vitamin D measurement within the specified 72-hour window, which could compromise data consistency and validity.

Sample size calculation

The required sample size was determined using G*Power version 3.1, based on a correlational model assessing the association between serum vitamin D levels and other clinical and demographic variables. A moderate effect size (*r* = 0.30) was chosen in line with previous literature on vitamin D and fracture-related outcomes. Using a two-tailed test, with a significance level (α) of 0.05 and statistical power (1 - β) of 0.80, the minimum required sample was calculated to be 119 participants. This estimate accounts for potential data loss and ensures adequate power to detect statistically meaningful associations between vitamin D levels and the contributing factors under investigation. [21]

Demographic and clinical data collection

Demographic and clinical data were collected through structured interviews, electronic medical records, and clinical examinations. The variables recorded included age, gender, body mass index (BMI), blood pressure levels, smoking status, seasonal variation, history of fractures (excluding hip fractures), current diet, use of mobility aids, family history of osteoporosis, sunlight exposure (UVB exposure), and calcium and vitamin D supplementation taken within the last month. Additionally, the study documented any relevant comorbid conditions. This comprehensive data collection was critical for identifying potential confounders and understanding how these factors might influence vitamin D levels and hip fracture risk in the elderly population.

Assessment of vitamin D levels and relevant biochemical and hematological biomarkers

The primary exposure variable, vitamin D levels, was assessed by measuring serum 25(OH)D concentrations through blood samples collected from participants and analyzed using the electrochemiluminescence immunoassay (CLIA) on a Roche Cobas e411 (Roche Diagnostics GmbH, Mannheim, Germany) analyzer. Vitamin D levels were categorized according to clinical guidelines as deficient (<20 ng/mL), insufficient (20-29 ng/mL), and sufficient (≥30 ng/mL). In addition to vitamin D, other relevant biochemical and hematological biomarkers were also measured. Biochemical parameters were analyzed using the Roche Cobas C111, while hematological parameters were assessed using the Horiba Yumizen H550 (HORIBA ABX SAS, Montpellier, France) analyzer. These included serum calcium, C-reactive protein (CRP), uric acid, hemoglobin, total white blood cell (WBC) count, neutrophil count, lymphocyte count, monocyte count, eosinophil count, basophil count, platelet count, alanine aminotransferase (ALT), aspartate aminotransferase (AST), alkaline phosphatase (ALP), serum albumin, serum bilirubin, serum creatinine, and blood urea levels. These biomarkers were crucial in evaluating the participants' overall health, inflammatory status, liver function, and kidney function, providing further insights into factors influencing vitamin D levels and fracture risk.

Statistical analysis

All statistical analyses were conducted using R (version 4.4.2) for data analysis and visualization, while Python libraries (Scikit-Learn, Seaborn, Matplotlib) were employed for additional graphical representations. The Shapiro-Wilk test was used to assess data normality. Continuous variables were presented as mean ± standard deviation (SD), while categorical variables were summarized with frequencies and percentages. Analysis of variance (ANOVA) was applied for comparing vitamin D levels across multiple groups, and for non-parametric data, the Mann-Whitney U test was used. In cases where these tests showed significant differences, decision tree analysis using the Classification and Regression Trees (CART) algorithm was performed to examine how the identified variables could predict vitamin D levels, with the Gini index indicating the importance of each predictor. Boxplots were used to compare vitamin D levels across variables such as age categories, dietary habits, blood pressure categories, fracture history, and vitamin D supplementation dosage, highlighting central tendency, spread, and outliers. Violin plots with boxplots were employed to show the distribution of vitamin D levels across BMI categories, seasonal variations, and fracture history, providing a comprehensive view of the data. Raincloud plots visualized the distribution of vitamin D levels about family history of osteoporosis, use of mobility aids, gender, smoking history, sunlight exposure, and comorbidities, combining boxplots, density plots, and individual data points to identify significant differences across groups. Logistic regression was conducted to evaluate the impact of comorbidities on vitamin D levels, with the results shown in the logistic regression coefficients plot. Decision tree models explored the influence of BMI, family history of osteoporosis, mobility aids, sunlight exposure, vitamin D supplementation, and calcium supplementation on vitamin D levels, with the Gini index assessing the importance of each predictor. Scatter plots were used to explore correlations between vitamin D levels and various biomarkers, and these correlations were quantified, with biomarkers ranked by their strength of association with vitamin D levels. Cluster analysis categorized patients into low, moderate, and high-risk groups based on their biomarker profiles and vitamin D levels. A *P*-value of <0.05 was considered statistically significant.

Ethical considerations

The study was carried out in full compliance with ethical guidelines approved by the Institutional Review Board (IRB). All participants provided written informed consent before participation, ensuring they understood the study's purpose, procedures, and potential risks. Confidentiality was maintained throughout, with all data anonymized and securely stored. The research did not impose any additional financial or medical burdens on participants, as the laboratory investigations were funded.

## Results

This study involved 119 elderly patients (mean age 67.53 ± 10.51 years, 69 males, 57.98%, and 50 females, 42.02%). The average BMI was 28.27 ± 5.67, with 59 patients (49.58%) reporting a history of smoking and 58 patients (48.74%) having a family history of osteoporosis. Mobility impairments were common, with 59 patients (49.58%) using aids. Vitamin D supplementation was taken by 68 patients (57.14%), with an average intake of 505.99 ± 284.26 IU/day, while 44 patients (36.97%) took calcium supplements, averaging 685.71 ± 223.15 mg/day. Comorbidities included diabetes in 76 patients (63.87%), asthma in 25 (21.01%), and hypertension in 21 (17.65%). Dietary habits varied, with 34 patients (28.57%) following a vegetarian diet. Regarding fractures, 64 patients (53.78%) had no previous fractures, and 55 patients (46.22%) had various types, including arm (16.81%), leg (10.92%), and wrist (9.24%) fractures. Most patients had limited sunlight exposure (5.63 ± 2.79 hours per week), possibly affecting vitamin D synthesis (Table 1).

**Table 1:** Patients' demographic, lifestyle, and health characteristics in a study on hip fracture risk and vitamin D levels. Data are presented as mean and standard deviation or frequency and percentages.

Characteristics	Values
Total no. of patients	119 (100)
Age (Years)	67.53 ± 10.51
Gender	
Female	50 (42.02%)
Male	69 (57.98%)
Body mass index (BMI)	28.27 ± 5.67
Blood pressure (mmHg)	
Systolic	138.68 ± 24.77
Diastolic	91.24 ± 17.09
History of smoking	59 (49.58%)
Family history of osteoporosis	58 (48.74%)
Use of mobility aids	59 (49.58%)
Vitamin D supplement intake (IU/day) during the previous month	
Not taking	51 (42.86%)
Taking	505.99 ± 284.26
Calcium supplement intake (mg/day) over the past month	
Not taking	44 (36.97%)
Taking	685.71± 223.15
Diet	
Vegetarian	34 (28.57%)
Westernized diet	30 (25.21%)
Traditional Pakistani diet	28 (23.53%)
Mixed diet	27 (22.69%)
Previous history of fracture other than hip in lifetime	
No history of fractures	64 (53.78%)
Arm	20 (16.81%)
Leg	13 (10.92%)
Wrist and ankle	11 (9.24%)
Spinal	05 (4.20%)
Ankle	04 (3.36%)
Seasons	
Fall	35 (29.41%)
Spring	34 (28.57%)
Winter	25 (21.01%)
Summer	25 (21.01%)
Sunlight exposure (UVB exposure) (per week)	5.63 ± 2.79
Comorbidities	
None	6 (5.04%)
Diabetes	76 (63.87%)
Asthma	25 (21.01%)
Gout	21 (17.65%)
Hypertension	21 (17.65%)
Chronic kidney disease (CKD)	18 (15.13%)
Hepatitis C	10 (8.40%)
Hepatitis B	10 (8.40%)
Thyroid disorder	06 (5.04%)
Chronic obstructive pulmonary disease (COPD)	05 (4.20%)
Stroke	03 (2.52%)
Epilepsy	03 (2.52%)
Anxiety	02 (1.68%)
Depression	02 (1.68%)
Parkinson’s disease	01 (0.84%)
Alzheimer’s disease	01 (0.84%)

In patients with hip fractures, biochemical and hematological profiles reveal patterns associated with poor bone health, inflammation, and systemic stress. The mean serum vitamin D level was 15.01 ± 5.51 ng/mL, indicating a significant deficiency, well below the recommended threshold of 20-30 ng/mL. Serum calcium levels were suboptimal at 7.38 ± 0.59 mg/dL, potentially contributing to bone demineralization. C-reactive protein (CRP) was elevated at 50.24 ± 16.36 mg/L, indicating inflammation that may hinder bone healing. Uric acid levels were moderately elevated at 5.75 ± 1.53 mg/dL, possibly linked to dietary factors or comorbidities like gout. Hemoglobin levels averaged 10.1 ± 1.91 g/dL, suggesting mild to moderate anemia, while white blood cell (WBC) count was high at 16,695.56 ± 4,768.98 /µL, with increased neutrophils (10,852.12 ± 3,099.84 /µL) and lymphocytes (5,008.67 ± 1,430.69 /µL), indicating systemic inflammation or infection. Platelet counts were elevated at 445,280.59 ± 56,792.64 /µL, possibly reactive to inflammation or anemia. Liver function tests showed mildly elevated ALT and AST (42.6 U/L) and significantly raised alkaline phosphatase (388.35 ± 119.59 U/L), often linked to bone turnover. Serum albumin was 3.8 ± 0.34 g/dL, within the normal range but low, and bilirubin was near the upper limit at 1.18 ± 0.2 mg/dL. Renal markers, including serum creatinine (1.35 ± 0.99 mg/dL) and blood urea (47.38 ± 30.49 mg/dL), suggested varying levels of renal impairment or dehydration (Table 2).

**Table 2:** Biochemical and hematological profiles of all patients with hip fractures. Data are presented as mean and standard deviation.

Characteristics	Values
Vitamin D levels	15.01 ± 5.51
Serum calcium levels	7.38 ± 0.59
Serum C-reactive protein levels	50.24 ± 16.36
Uric acid levels	5.75 ± 1.53
Hemoglobin levels	10.1 ± 1.91
Total white blood cell count	16695.56 ± 4768.98
Neutrophils count	10852.12 ± 3099.84
Lymphocytes count	5008.67 ± 1430.69
Monocytes count	834.78 ± 238.45
Eosinophils count	500.87 ± 143.07
Basophils count	333.91 ± 95.38
Platelets count	445280.59 ± 56792.64
Alanine aminotransferase levels	42.64 ± 8.9
Aspartate aminotransferase levels	42.68 ± 10.33
Alkaline phosphatase levels	388.35 ± 119.59
Serum albumin levels	3.8 ± 0.34
Serum bilirubin levels	1.18 ± 0.2
Serum creatinine levels	1.35 ± 0.99
Blood urea levels	47.38 ± 30.49

**Figure 1:**
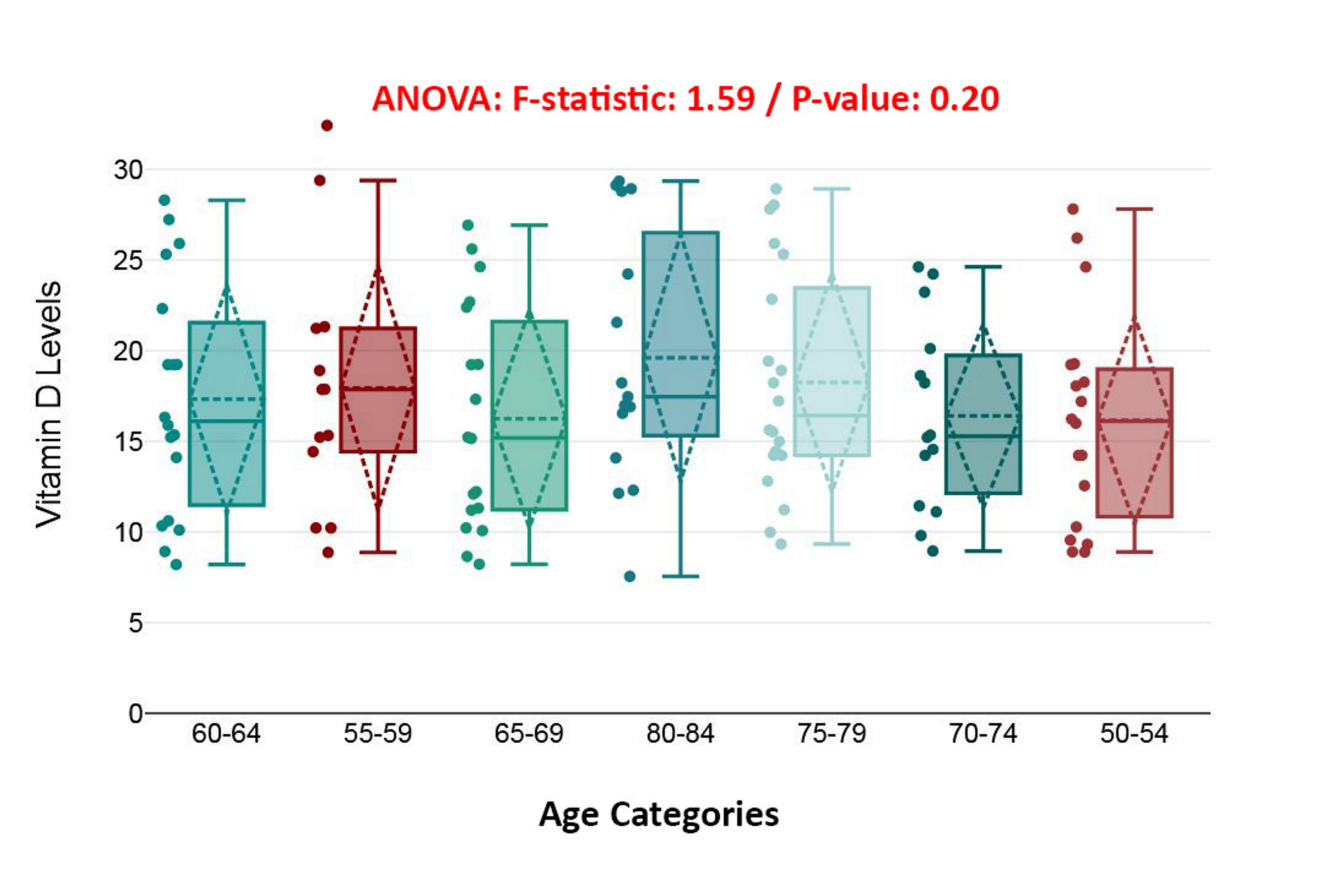
Age variation of vitamin D levels in elderly patients with hip fractures.

The boxplot displays the distribution of vitamin D levels across age categories (50-54 to 80-84 years) among patients with hip fractures. Although there are slight visual differences in the median and spread between groups, the one-way analysis of variance (ANOVA) test showed an *F*-statistic of 1.59 and a *P*-value of 0.20, indicating no statistically significant differences in vitamin D levels across age groups. This suggests that age is not a major factor influencing vitamin D status in this population, and the observed variations are likely due to chance rather than an age-related trend (Figure 1).

**Figure 2:**
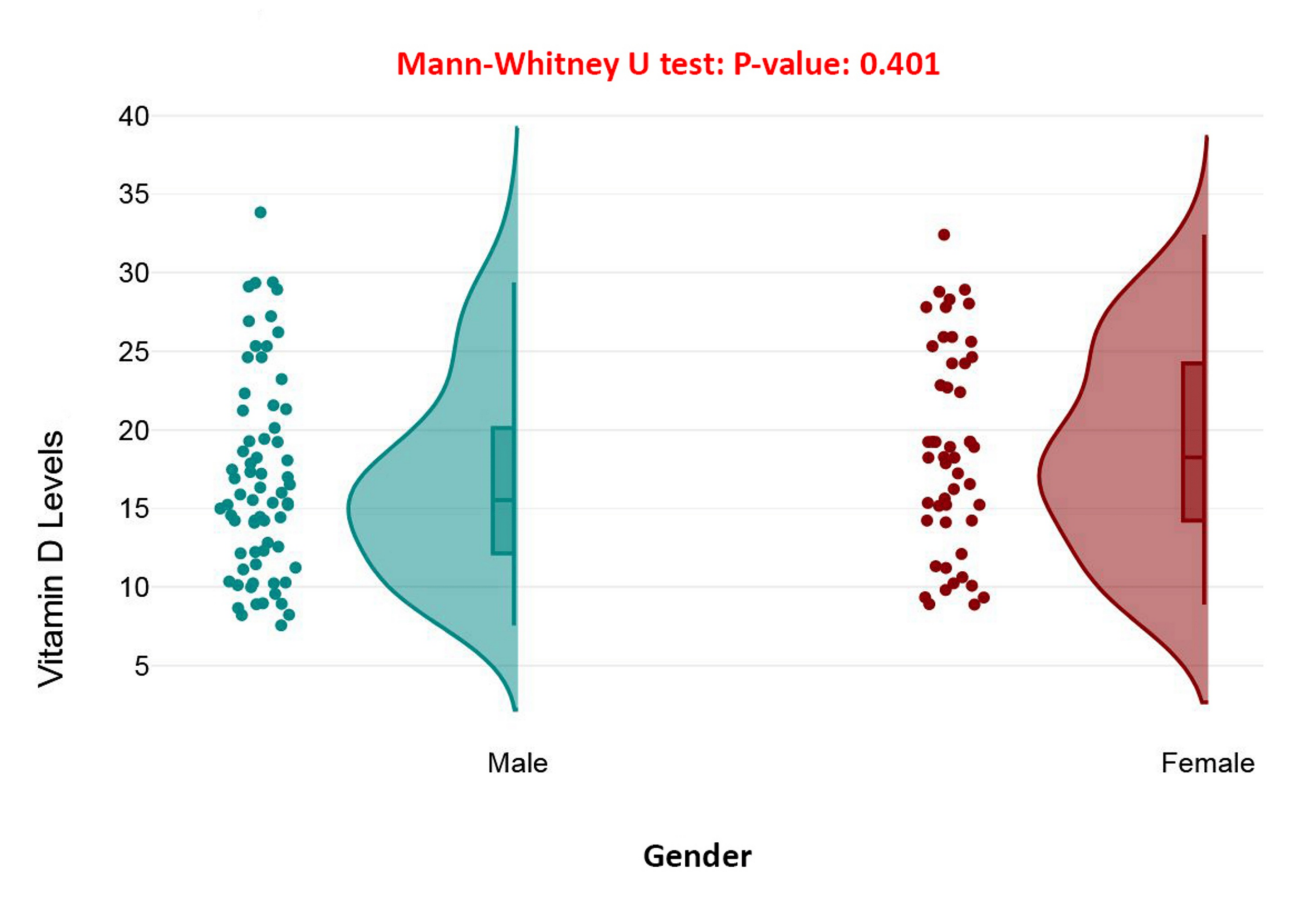
Comparison of vitamin D levels by gender in patients with hip fractures.

The raincloud plot compares vitamin D levels between male and female patients with hip fractures, showing similar distributions for both genders, with levels primarily ranging from 10 to 25 ng/mL. The Mann-Whitney U test yielded a *P*-value of 0.401, indicating no statistically significant difference in vitamin D levels between males and females. This suggests that gender does not influence vitamin D status in this cohort, and both sexes exhibit comparable levels of deficiency or insufficiency (Figure 2).

**Figure 3:**
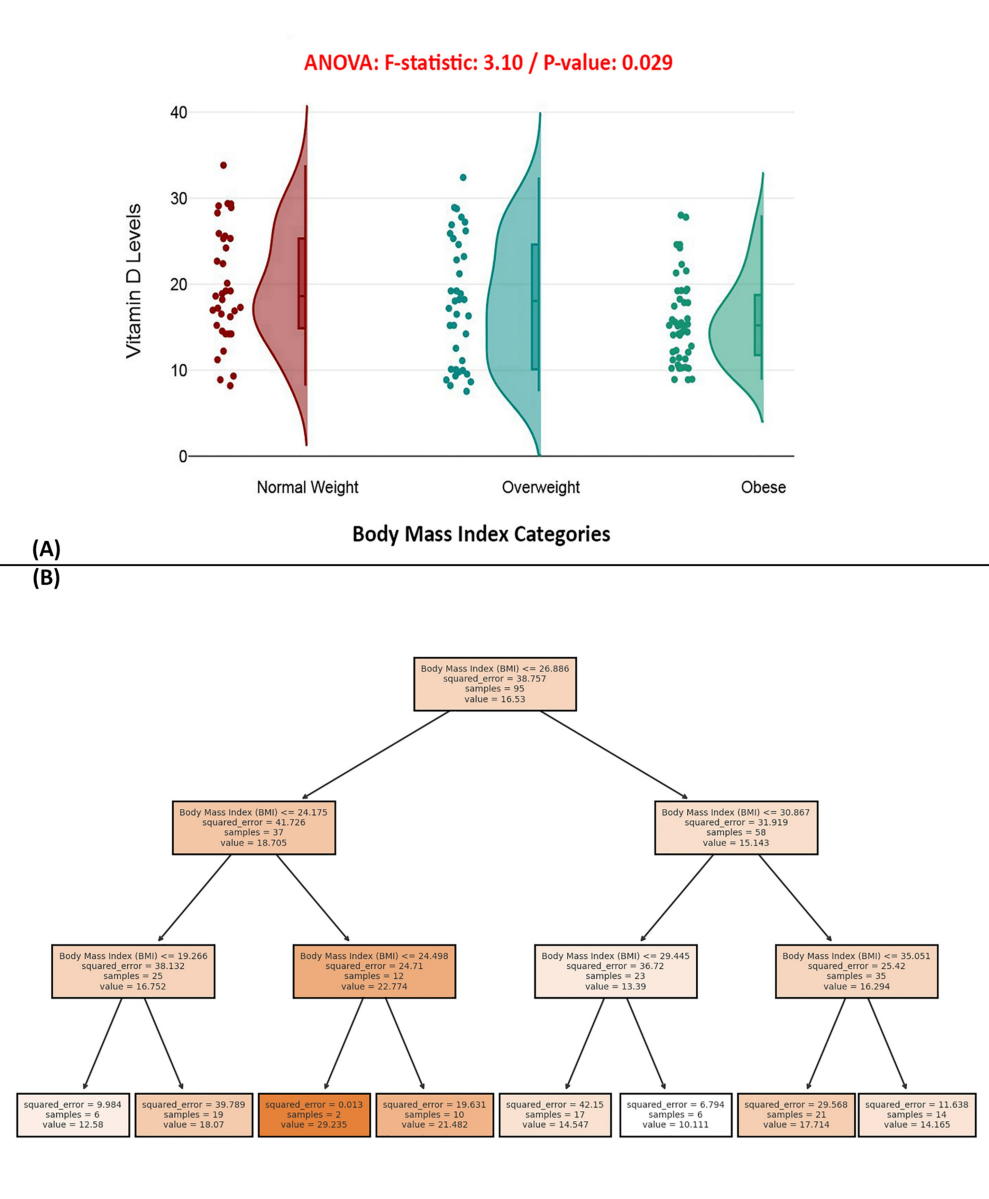
BMI as a significant predictor of vitamin D levels in patients with hip fractures. (A) Raincloud plot of vitamin D levels across BMI categories. (B) Decision tree model predicting vitamin D levels based on BMI. BMI, body mass index

The raincloud plot in Figure 3A indicates that patients with normal weight generally have higher vitamin D levels than overweight and obese individuals. The ANOVA test produced an *F*-statistic of 3.10 and a *P*-value of 0.029, indicating a statistically significant difference in vitamin D levels across BMI categories. This finding is supported by the decision tree model Figure 3B, which shows that lower BMI is consistently linked to higher vitamin D levels, while higher BMI correlates with lower levels. These results suggest that BMI is a significant predictor of vitamin D deficiency, with obesity potentially affecting vitamin D bioavailability or metabolism (Figure 3).

**Figure 4:**
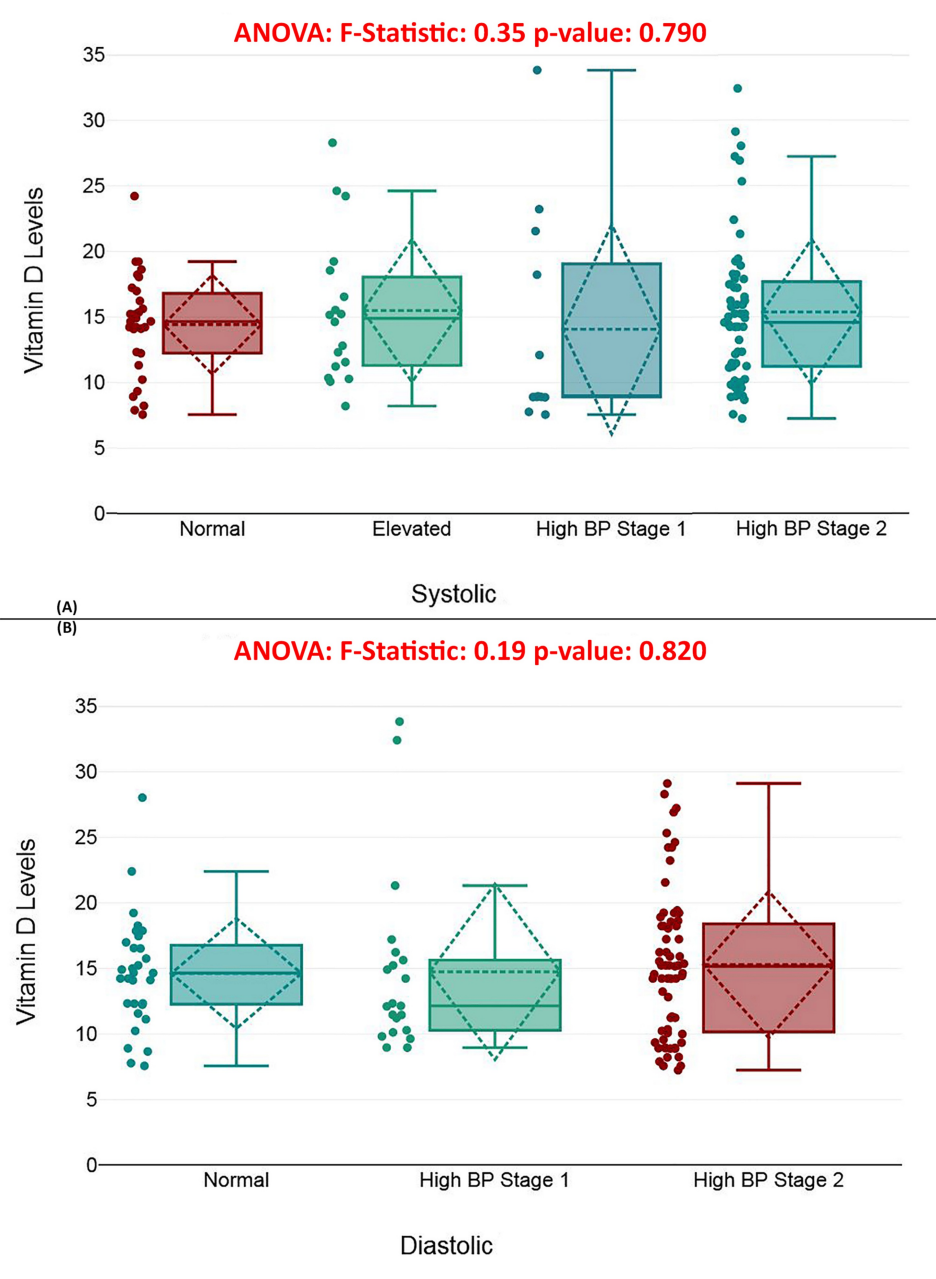
Blood pressure and vitamin D levels in patients with hip fractures. (A) Comparison of vitamin D levels across systolic blood pressure categories. (B) Comparison of vitamin D levels across diastolic blood pressure categories. Categories of blood pressure: (1) Systolic pressure categories (in mmHg): normal, <120; elevated, 120-129; high blood pressure stage 1, 130-139; high blood pressure stage 2, 140+. (2) Diastolic pressure categories (in mmHg): normal, <80; high blood pressure stage 1, 80-89; high blood pressure stage 2, 90+.

Figure 4A presents a boxplot comparing vitamin D levels across systolic blood pressure categories (normal, elevated, high BP stage 1, high BP stage 2), while Figure 4B does the same for diastolic blood pressure categories. Despite minor visual differences, ANOVA revealed no significant variation in vitamin D levels across systolic (*F* = 0.35, *P* = 0.790) or diastolic (*F* = 0.19, *P* = 0.820) blood pressure groups. These findings suggest that blood pressure, whether systolic or diastolic, does not significantly affect vitamin D levels in this cohort, and hypertension does not appear to be a major factor influencing vitamin D status regarding hip fracture risk (Figure 4).

**Figure 5:**
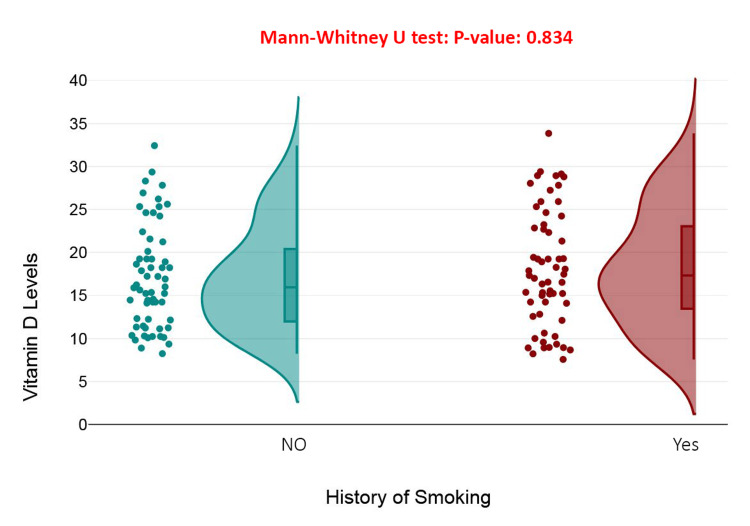
Difference in vitamin D levels based on smoking history among patients with hip fractures.

The raincloud plot compares vitamin D levels between patients with and without a history of smoking. Despite slight visual differences in distribution, the Mann-Whitney U test yielded a *P*-value of 0.834, indicating no statistically significant difference in vitamin D levels between smokers and non-smokers. Both groups exhibit similar median values and variability, suggesting that smoking history does not influence serum vitamin D concentrations in this cohort of elderly patients with hip fractures (Figure 5).

**Figure 6:**
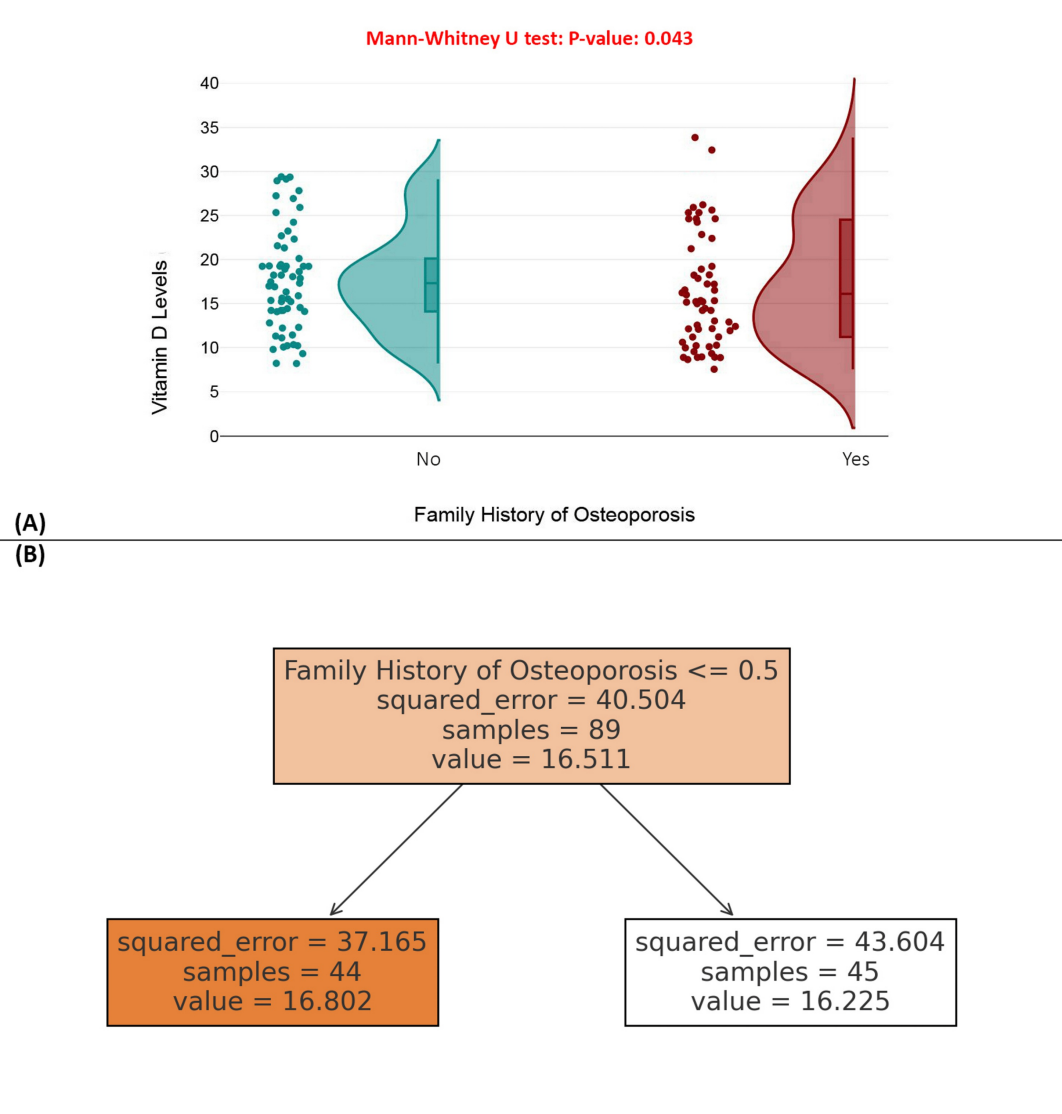
Family history of osteoporosis and vitamin D level variation among patients with hip fractures. (A) Raincloud plot of vitamin D levels by family history of osteoporosis. (B) Decision tree model predicting vitamin D levels based on family history of osteoporosis.

Figure 6A presents a raincloud plot comparing vitamin D levels between those with and without a family history of osteoporosis. The Mann-Whitney U test showed a *P*-value of 0.043, indicating a statistically significant difference, with individuals having a family history exhibiting slightly lower median vitamin D levels. Figure 6B features a decision tree model that confirms the split based on family history but shows similar predicted mean vitamin D levels for both groups (16.80 vs. 16.22), suggesting that while the association is statistically significant, its clinical significance may be limited. Overall, this analysis highlights the family history of osteoporosis as a relevant factor influencing vitamin D status (Figure 6).

**Figure 7:**
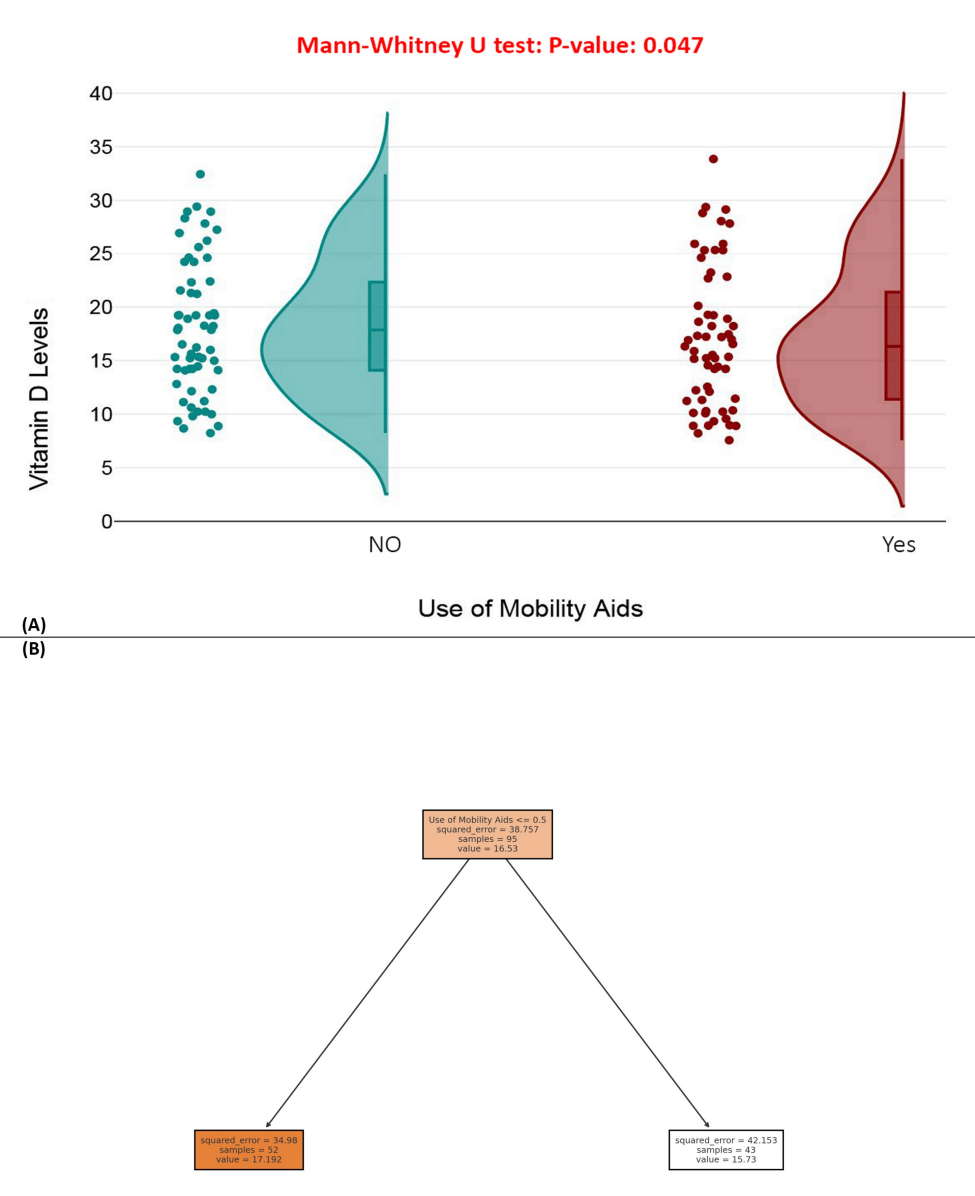
Use of mobility aids and vitamin D levels. (A) Raincloud plot of vitamin D levels by use of mobility aids. (B) Decision tree model predicting vitamin D levels based on use of mobility aids.

Figure 7A presents a raincloud plot comparing vitamin D levels between patients who use mobility aids and those who do not. The Mann-Whitney U test yielded a *P*-value of 0.047, indicating a statistically significant difference, with mobility aid users generally having lower serum vitamin D concentrations. Figure 7B displays a decision tree model, distinguishing between users and non-users of mobility aids, with predicted mean vitamin D levels of 17.19 ng/mL for non-users and 15.73 ng/mL for users. While the numerical difference is modest, both visual and statistical results suggest that limited mobility may contribute to lower vitamin D levels, likely due to reduced sun exposure or physical activity (Figure 7).

**Figure 8:**
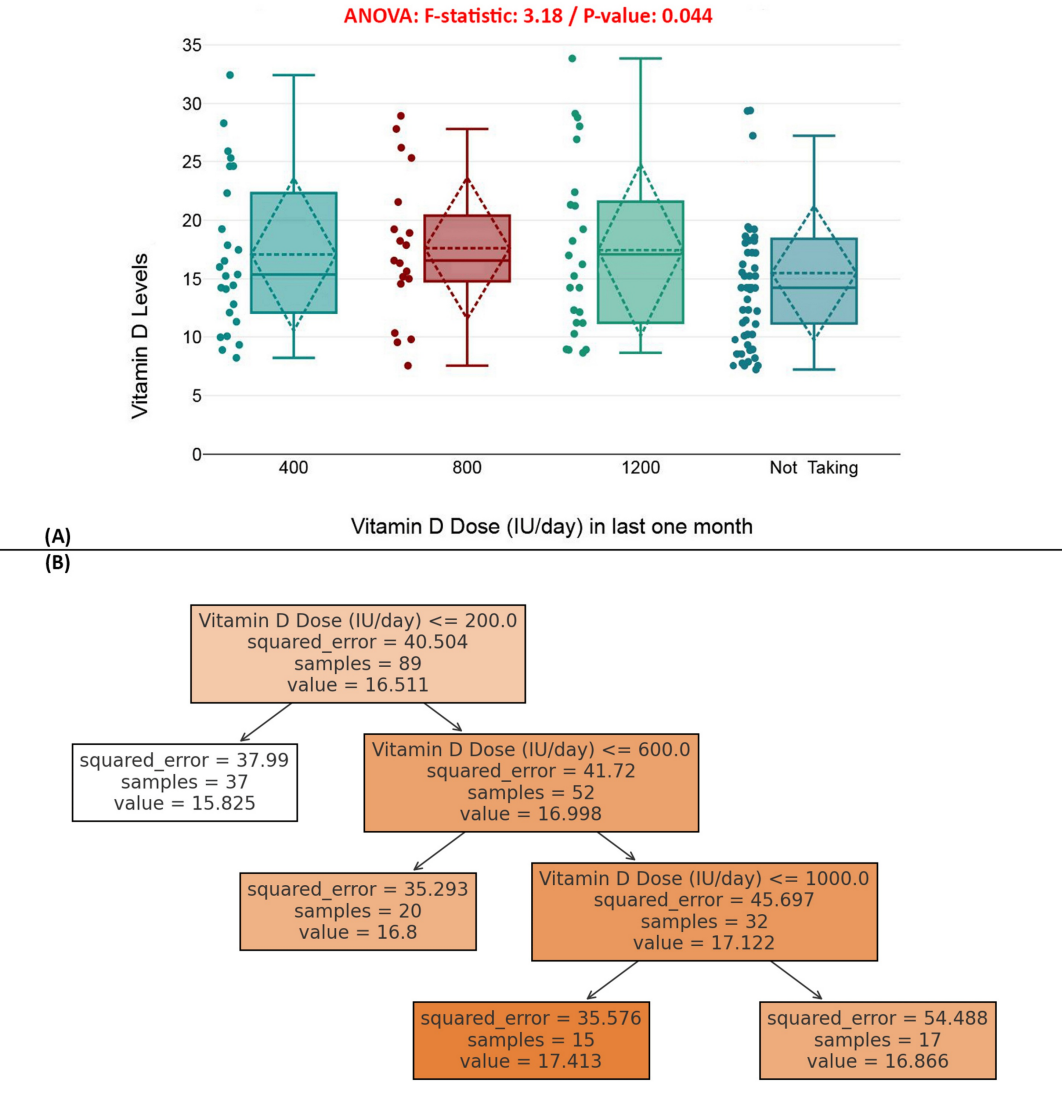
Impact of vitamin D dosage on serum vitamin D levels. (A) Boxplot showing the distribution of serum vitamin D levels by recent supplementation dosage. (B) Decision tree model for predicting serum vitamin D levels based on daily dosage.

Figure 8A presents a boxplot comparing serum vitamin D levels across supplementation groups (400 IU, 800 IU, 1200 IU per day, and non-users), revealing a statistically significant difference (ANOVA *F* = 3.18, *P* = 0.044), indicating that dosage has a notable, albeit modest, effect on serum levels. Notably, individuals taking 800 IU daily showed more concentrated values with lower variability, while non-users had broader ranges and generally lower levels. Figure 8B illustrates a regression decision tree model that segments participants by dosage thresholds to predict vitamin D levels. It shows that higher doses (>600 IU/day) slightly improve predicted vitamin D levels, though variability persists. The tree’s shallow hierarchy suggests that vitamin D dosage alone only modestly explains serum levels, with other factors such as absorption, comorbidities, and sun exposure likely influencing the results (Figure 8).

**Figure 9:**
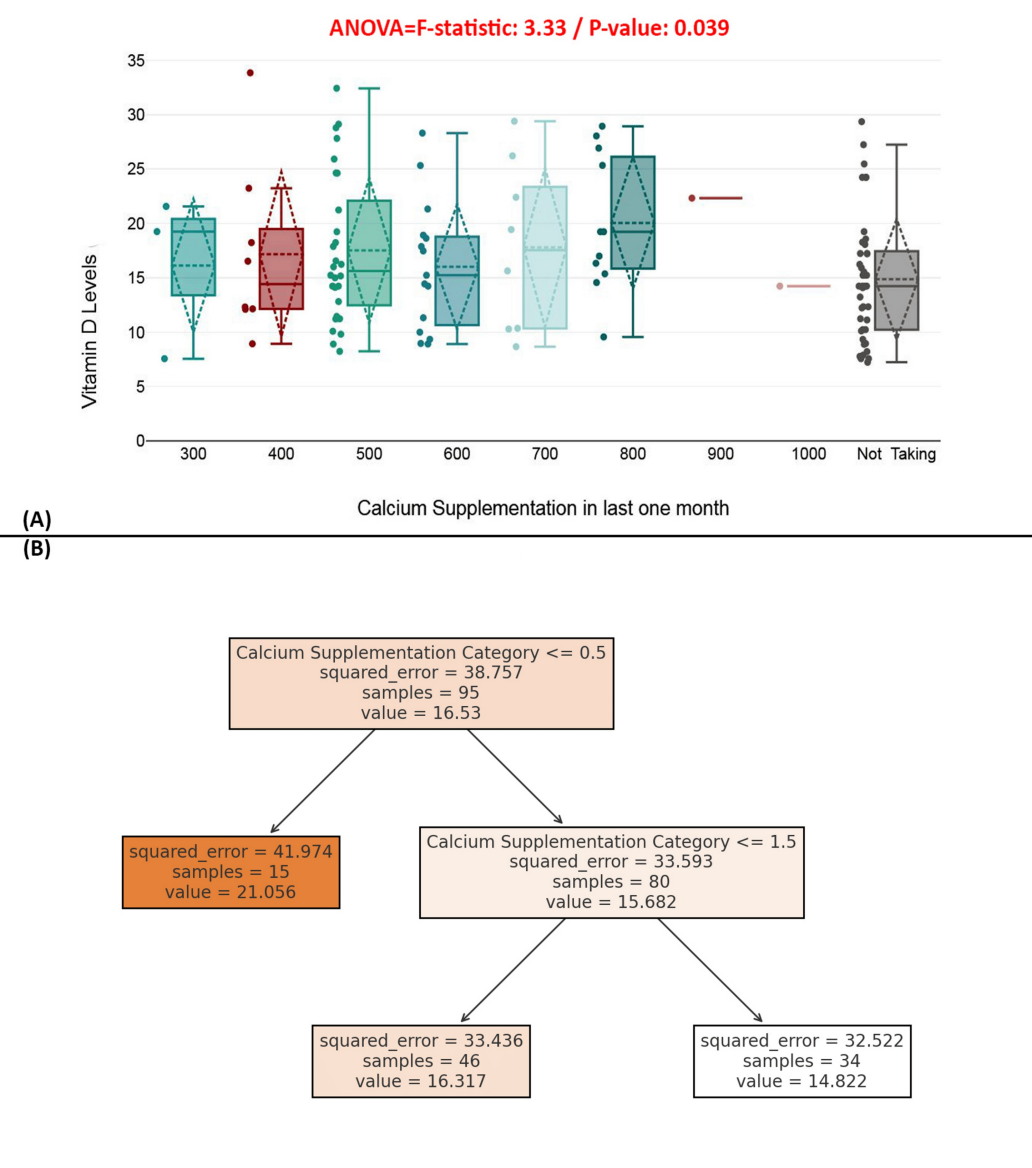
Relationship between calcium supplementation and serum vitamin D levels. (A) Boxplot showing the distribution of serum vitamin D levels by calcium supplementation dosage. (B) Decision tree regression model predicting serum vitamin D levels based on calcium supplementation.

Figure 9A presents a boxplot comparing vitamin D levels across different calcium intake categories over the past month, including a non-supplementing group. The ANOVA test showed a statistically significant difference (*F* = 3.33, *P* = 0.039), suggesting that calcium intake may modestly influence vitamin D levels. Higher calcium supplementation appears to be associated with slightly higher and less variable vitamin D levels, while non-users show lower median values and wider dispersion. Figure 9B displays a regression decision tree that uses calcium supplementation categories to predict vitamin D levels. The tree indicates that lower supplementation (≤0.5) is linked with higher predicted vitamin D levels in a small subset (value = 21.056), contrasting the general trend observed in the boxplot. Overall, while calcium supplementation is mildly associated with vitamin D status, the decision tree suggests that the relationship is not strictly linear, indicating other factors likely influence serum vitamin D levels (Figure 9).

**Figure 10:**
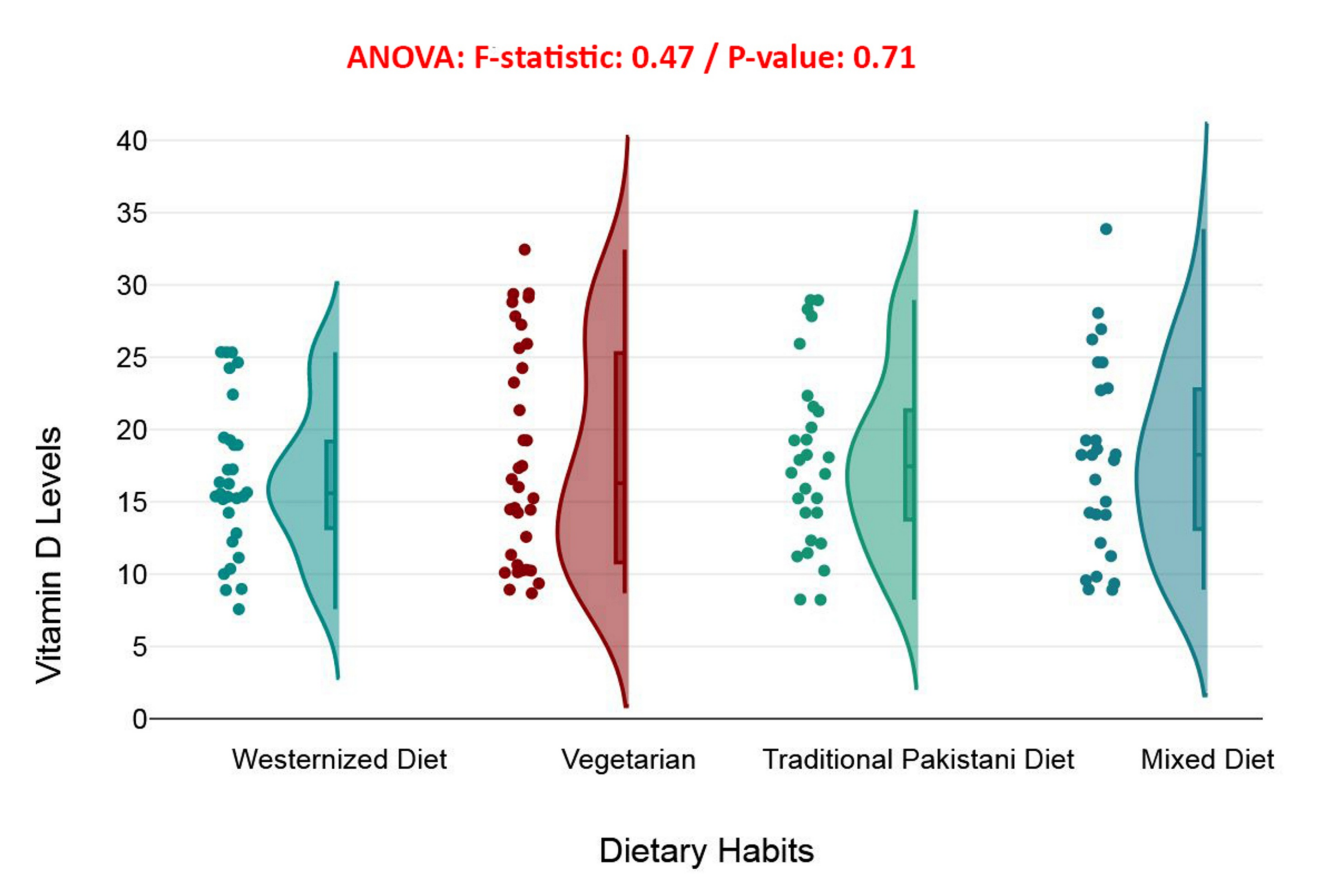
Association between dietary habits and serum vitamin D levels.

This raincloud plot visualizes the distribution of serum vitamin D levels across four dietary habit categories: Westernized diet, vegetarian, traditional Pakistani diet, and mixed diet. Although there are visible differences in distribution shapes and outliers, the ANOVA test produced an *F*-statistic of 0.47 with a non-significant *P*-value of 0.71, indicating no statistically significant variation in vitamin D levels among these dietary groups. Each category shows a similar median and interquartile range, suggesting that dietary habit alone is not a strong determinant of serum vitamin D status in this cohort. The overlapping density curves further highlight that visual differences in spread are not substantial enough to infer a dietary effect. These results suggest that factors such as sunlight exposure, supplementation, and metabolic health are more influential in determining vitamin D levels than dietary pattern alone (Figure 10).

**Figure 11:**
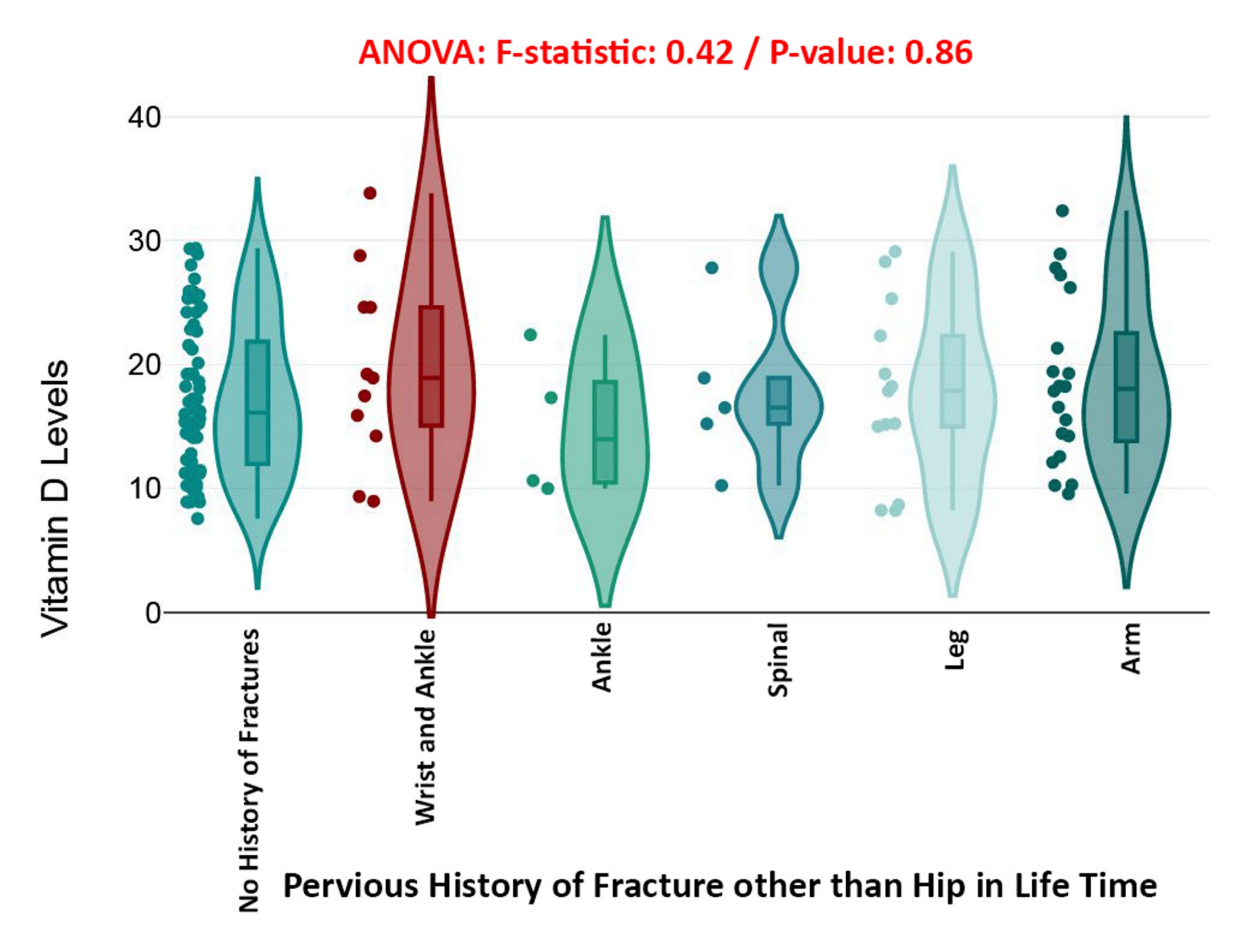
Serum vitamin D levels across different histories of non-hip fractures: boxplot with violin plot.

This boxplot within a violin plot illustrates the distribution of serum vitamin D levels among individuals with varying lifetime histories of fractures other than the hip, including categories such as wrist and ankle, ankle alone, spinal, leg, arm, and no fracture history. Despite visual differences in spread and density, ANOVA analysis revealed no significant differences across the groups (*F* = 0.42, *P* = 0.86). Median vitamin D levels were comparable among all fracture history groups, with overlapping interquartile ranges. Although the "Wrist and Ankle" and "Spinal" groups showed slightly wider variability and outliers, the overall similarity suggests that a history of non-hip fractures does not significantly impact current vitamin D status. These findings emphasize the importance of exploring other factors, such as supplementation, sun exposure, and comorbidities, when evaluating vitamin D levels in elderly patients (Figure 11).

**Figure 12:**
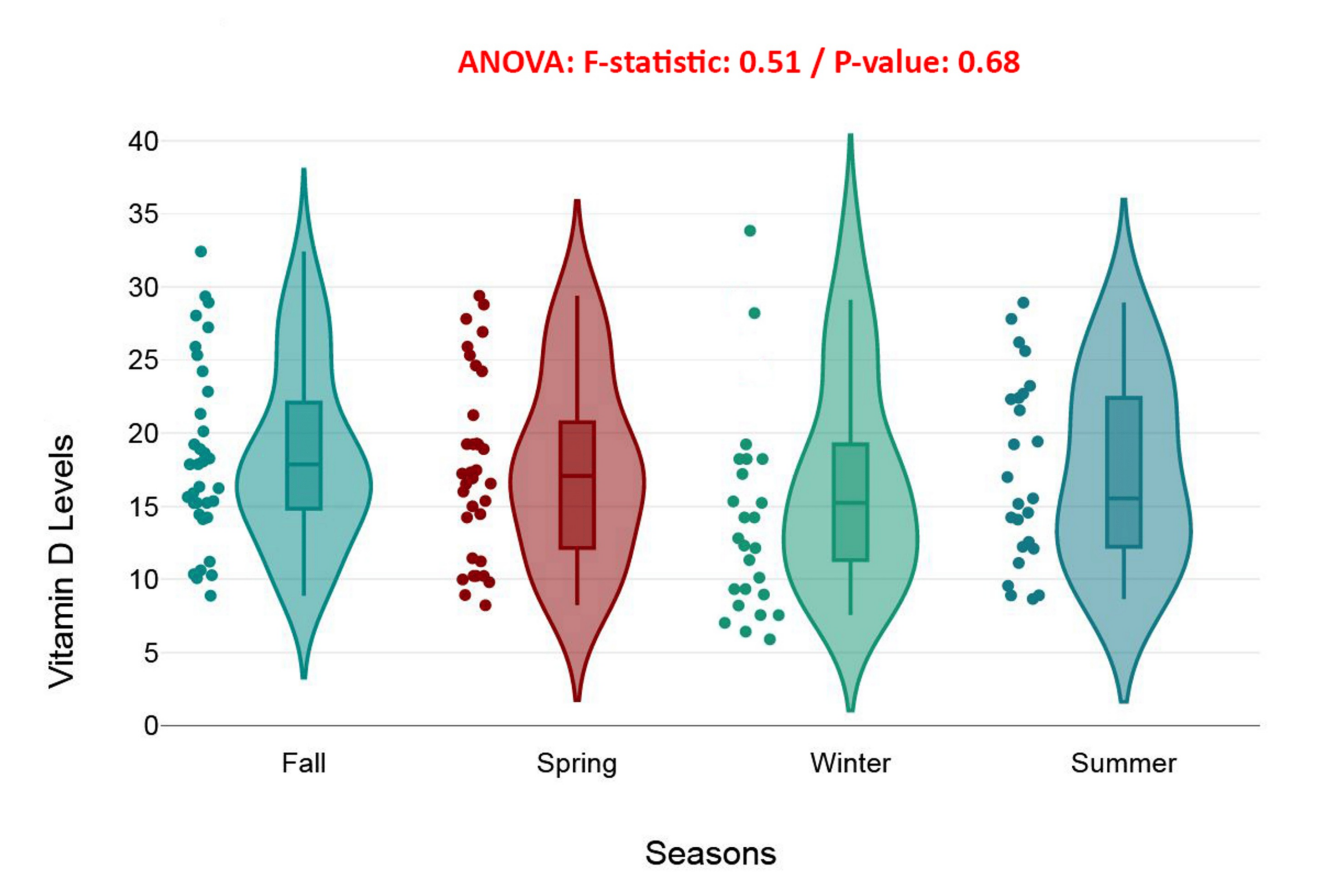
Seasonal variation in serum vitamin D levels: boxplot with violin plot analysis.

This boxplot with violin plot compares serum vitamin D levels across the four seasons: Fall, Spring, Winter, and Summer. Despite minor visual differences in spread and density, ANOVA results (*F* = 0.51, *P* = 0.68) indicate no statistically significant variation in vitamin D levels across seasons in this cohort. Median values and interquartile ranges were similar, with all seasons exhibiting comparable distribution shapes. While vitamin D levels appeared slightly more dispersed in Summer and Fall, these trends were not strong enough to suggest that seasonality is a meaningful determinant. This is unexpected, given that vitamin D synthesis is typically linked to sun exposure, which varies seasonally. The lack of significant differences may indicate that compensatory behaviors such as supplementation or more consistent indoor lifestyles in the elderly minimize the impact of seasonality (Figure 12).

**Figure 13:**
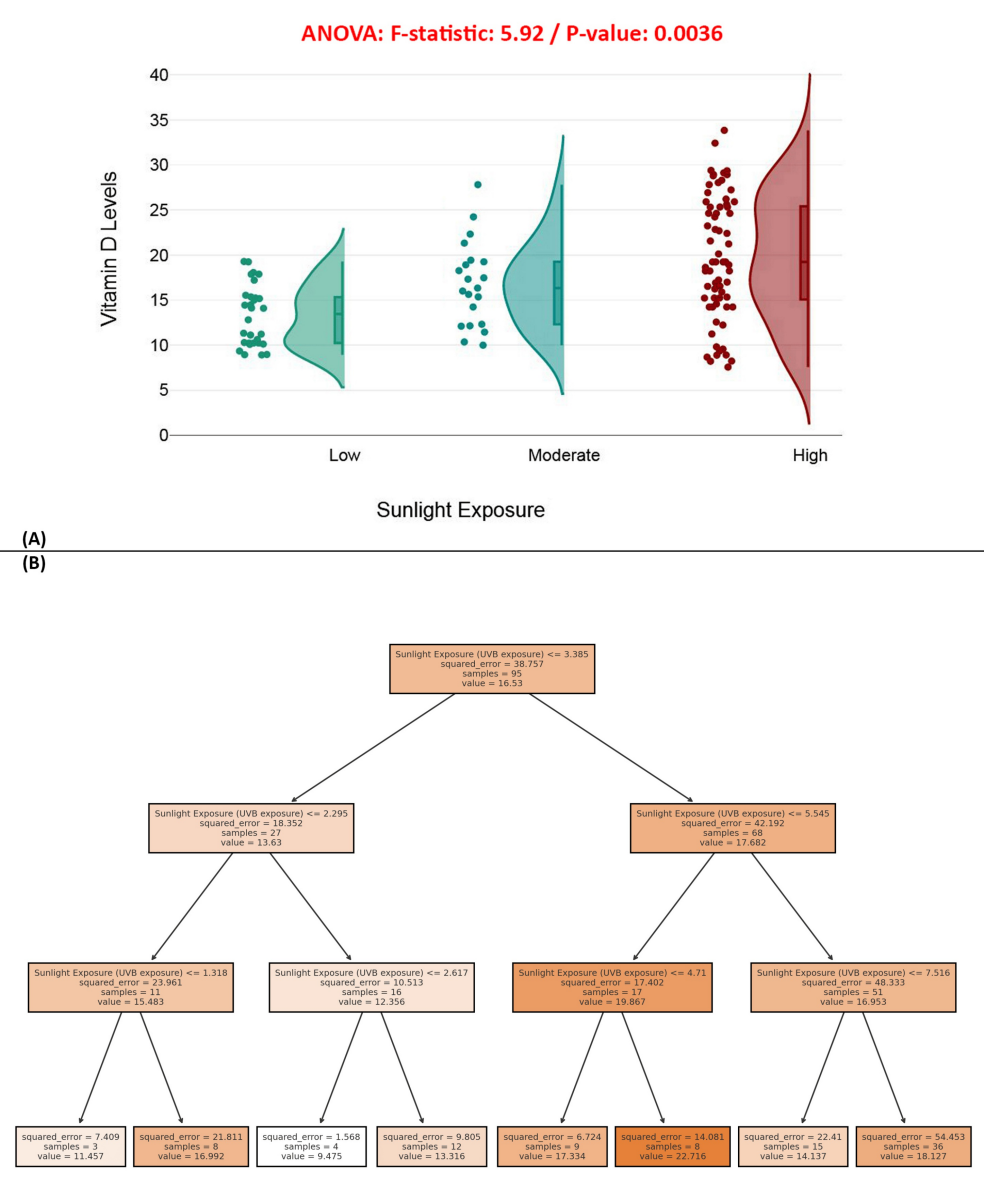
Impact of sunlight exposure on serum vitamin D levels. (A) Raincloud plot showing the distribution of serum vitamin D levels by sunlight exposure categories. (B) Decision tree model for predicting vitamin D levels based on UVB sunlight exposure. UVB exposure per week ranges for each category: Low: ≤ 2.295 Moderate: 2.295-5.545 High: ≥5.545

Figure 13A, a raincloud plot, compares vitamin D levels across three sunlight exposure groups: low, moderate, and high. A statistically significant difference is observed (ANOVA *F* = 5.92, *P* = 0.0036), with individuals in the high-exposure group showing higher median and less variable vitamin D levels compared to the low and moderate groups. This supports the established biological link between UVB exposure and vitamin D synthesis. Figure 13B presents a regression decision tree model that further stratifies vitamin D levels based on continuous UVB exposure. The tree reveals that higher UVB exposure (>5.545) consistently predicts higher vitamin D levels, with a terminal node value peaking at 22.716. Lower exposures (<2.617) are associated with significantly reduced vitamin D values. The branching pattern highlights sunlight exposure as a strong, non-linear predictor of vitamin D status, surpassing other variables examined. This analysis emphasizes sunlight exposure as a key modifiable factor in maintaining adequate vitamin D levels, especially in elderly populations. Figure 13

**Figure 14:**
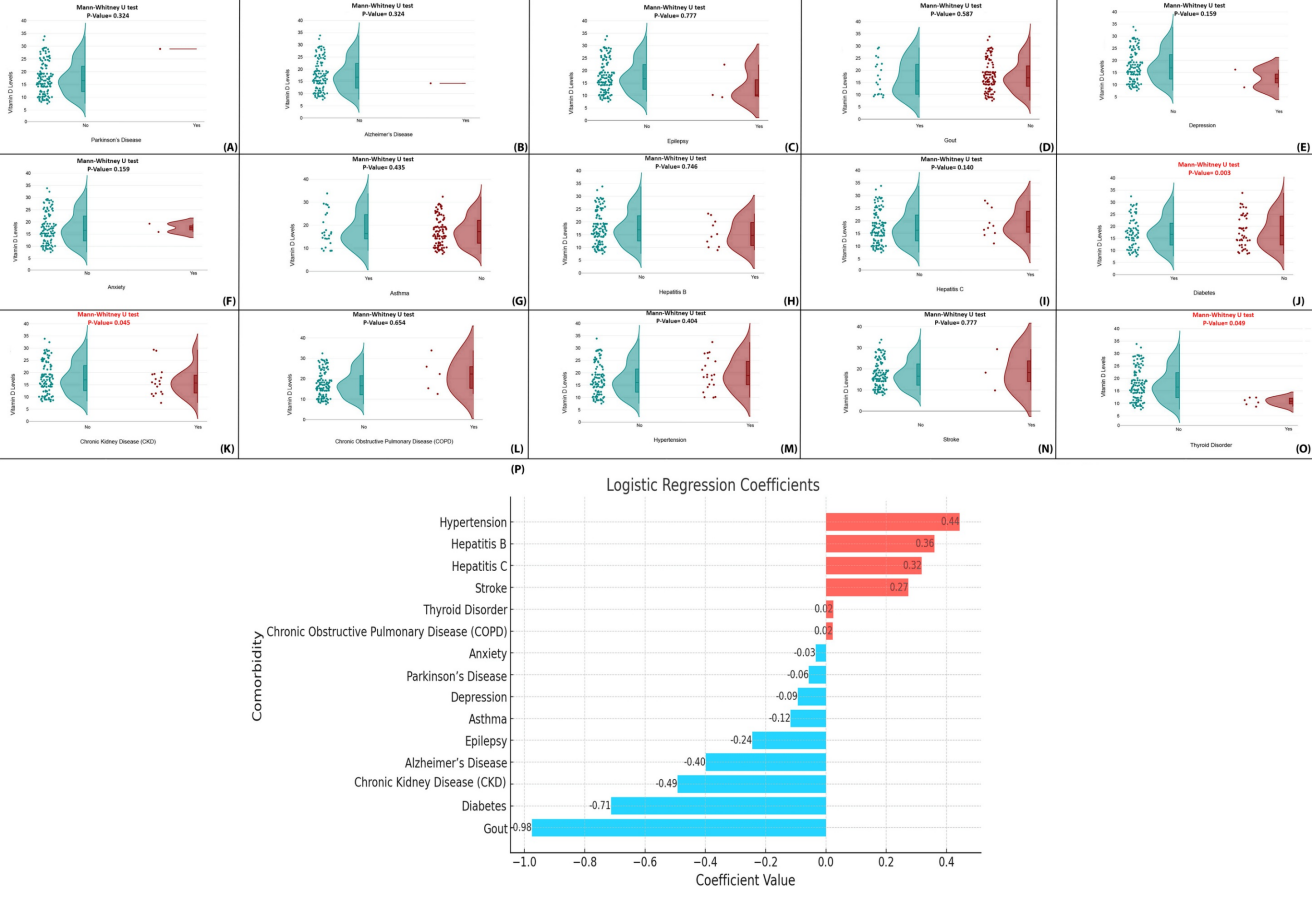
Influence of comorbidities on serum vitamin D levels. (A-O) Raincloud plots for individual comorbidities: Distribution of serum vitamin D levels in patients with and without specific comorbidities. (P) Logistic regression plot: Logistic regression coefficients indicating the influence of comorbidities on vitamin D deficiency.

Figures 14A-14O display raincloud plots comparing vitamin D levels in individuals with and without specific conditions, with Mann-Whitney U tests revealing significant differences (*P* < 0.05) for diabetes, chronic kidney disease (CKD), and thyroid disorders, all associated with lower vitamin D levels. Figure 14P shows a logistic regression coefficient plot identifying comorbidities most predictive of vitamin D insufficiency. Gout and diabetes exhibit the strongest negative associations (coefficients: -0.98 and -0.71), followed by CKD and Alzheimer’s. In contrast, hypertension, hepatitis B/C, and stroke show small positive associations, suggesting a slightly higher likelihood of normal vitamin D levels, possibly due to better medical supervision or supplementation. The consistency between univariate and regression findings strengthens the observed links. This analysis highlights that certain comorbidities, particularly metabolic and renal conditions, are significantly associated with lower vitamin D levels, emphasizing the need for targeted screening and supplementation in high-risk groups (Figure 14).

**Figure 15:**
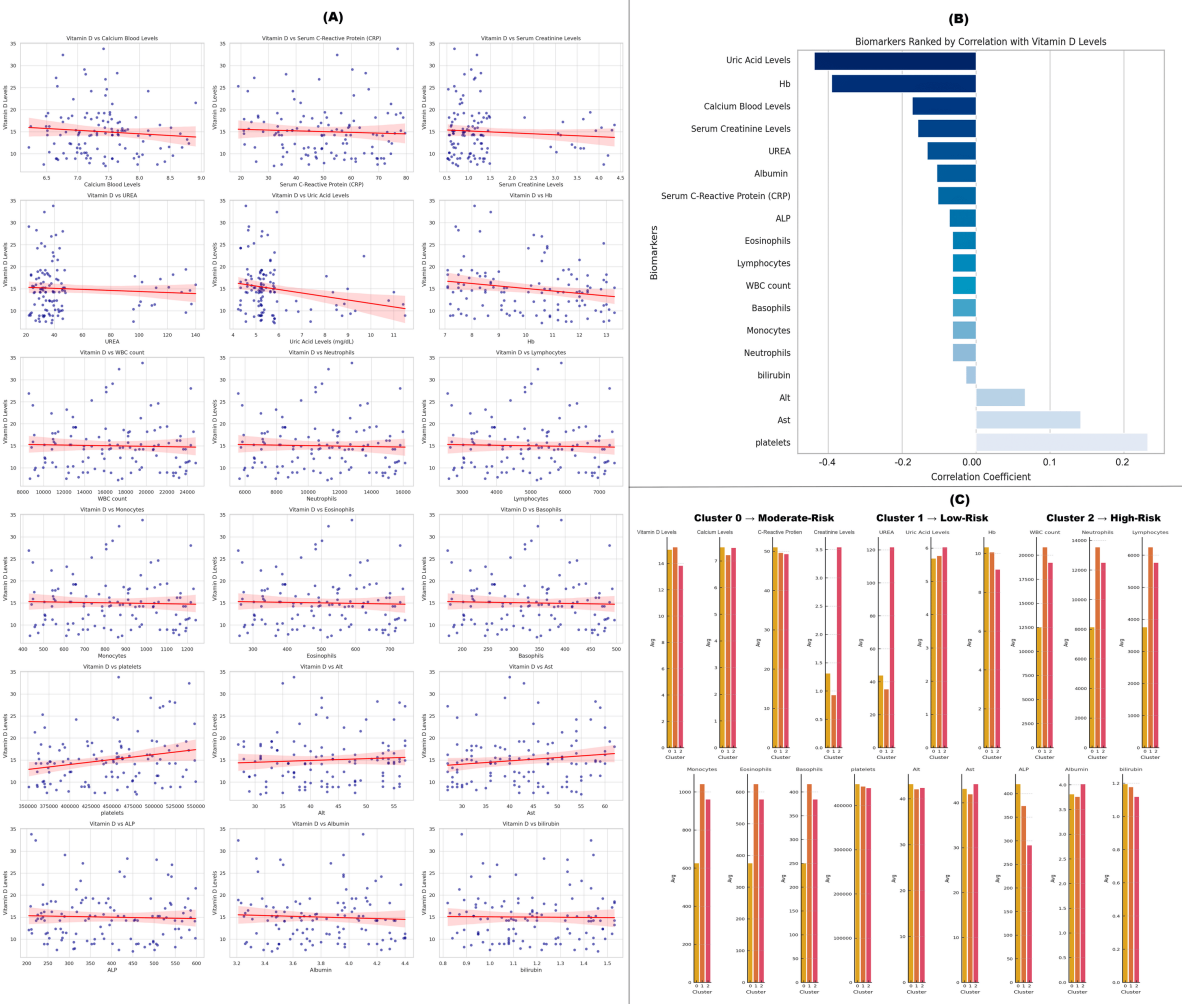
Association of clinical biomarkers with serum vitamin D levels. (A) Scatterplots of biomarkers: Bivariate relationships between serum vitamin D levels and individual clinical biomarkers. (B) Correlation ranking bar chart: Biomarkers ranked by Pearson correlation coefficient with serum vitamin D levels. (C) Cluster-based risk group analysis: Biomarker and vitamin D profiles across data-driven patient risk clusters. (1) Low risk: highest vitamin D levels (best among all). -Lowest CRP, urea, creatinine → low inflammation and good kidney health -High albumin and calcium → good nutrition and metabolic status 0: Moderate risk: Intermediate vitamin D levels -CRP, urea, creatinine values are worse than Cluster 1 but better than Cluster 2 -Albumin and other markers are not optimal but not the worst (2) High risk: lowest vitamin D levels -Highest CRP, urea, creatinine → inflammation and kidney stress -Lowest albumin and Hb → potential malnutrition or chronic disease

This composite figure presents a comprehensive analysis of how various clinical biomarkers relate to serum vitamin D levels and patient risk profiles. Figure 15A features scatterplots with regression lines showing the correlation between vitamin D and individual biomarkers. Negative trends are especially apparent with uric acid, serum creatinine, urea, and CRP, suggesting that higher levels of these markers-often associated with inflammation or renal dysfunction-correspond with lower vitamin D status. Figure 15B visualizes these associations via a bar chart ranking biomarkers by their correlation with vitamin D levels. Uric acid and hemoglobin exhibit the strongest negative and positive associations, respectively, while inflammatory markers and renal function indicators generally show inverse relationships. Figure 15C groups patients into three risk-based clusters (low, moderate, and high risk) using biomarker profiles. Cluster 2 (high risk) is characterized by elevated urea, creatinine, and CRP, and concurrently lower vitamin D levels, highlighting a clinically vulnerable subgroup. This integrative analysis supports the utility of vitamin D as a broader indicator of systemic health, linking it to both inflammatory and renal biomarkers and aiding in the stratification of patient risk based on biomarker clustering (Figure 15).

## Discussion

The relationship between serum vitamin D concentrations and the risk of hip fractures in geriatric populations has been extensively established in scientific literature, underscoring a pressing public health concern. This is particularly relevant given the multifaceted etiology of vitamin D deficiency and its deleterious effects on skeletal integrity. In the current investigation, the mean serum level of 25(OH)D among elderly participants was notably low, measured at 15.01 ± 5.51 ng/mL - substantially below the widely recognized threshold for sufficiency, generally defined between 20 and 30 ng/mL [22]. This finding is consistent with global epidemiological data that report a high prevalence of hypovitaminosis D among aging populations. The underlying causes have been attributed to a confluence of factors, including diminished dermal synthesis of vitamin D due to age-related changes, inadequate dietary intake, and reduced exposure to sunlight [23,24].

A critical component contributing to this deficiency appears to be limited ultraviolet B (UVB) exposure. The mean weekly UVB exposure among the study cohort was only 5.63 ± 2.79 hours, further compounding concerns regarding suboptimal vitamin D status [22]. Previous studies have robustly demonstrated a direct, statistically significant association between sunlight exposure and circulating vitamin D levels, a relationship reaffirmed in this study with a *P*-value of 0.0036 [24]. These findings accentuate the importance of developing public health policies aimed at promoting safe and effective sun exposure, particularly within elderly populations, who may be constrained by cultural, environmental, or physical limitations that restrict outdoor activities [25].

Interestingly, traditional demographic variables such as chronological age and biological sex were not found to be significant predictors of serum vitamin D status in the current sample. This conclusion was supported by both one-way ANOVA and Mann-Whitney U tests, which yielded non-significant outcomes. These results suggest that although age-related physiological changes can impair vitamin D synthesis and metabolism, other variables-such as behavioral patterns, physical activity levels, or concurrent medical conditions-may exert a more pronounced influence on serum concentrations [26]. Moreover, the absence of sex-based differences in vitamin D status could be attributed to similar patterns of supplement use and dietary intake across male and female participants in the cohort [27].

BMI emerged as a significant determinant of serum vitamin D levels, corroborating existing research that points to the sequestration of fat-soluble vitamins like vitamin D in adipose tissue, thus limiting their bioavailability [28]. In this study, a significant inverse association between BMI and serum 25(OH)D was observed (p = 0.029), highlighting the importance of weight management in addressing vitamin D deficiency among older adults [27]. Given the cohort's mean BMI of 28.27 ± 5.67, which falls within the overweight range, this finding underscores the interplay between adiposity and nutrient distribution, particularly in vulnerable populations [29].

Mobility status was another variable significantly associated with vitamin D levels. Approximately 49.58% of participants required assistive devices for ambulation, and their serum 25(OH)D concentrations were significantly lower than those of non-users (*P* = 0.047). The likely mechanisms involve decreased outdoor exposure and reduced physical activity, both of which are vital for endogenous vitamin D synthesis [25]. Although the absolute reduction in serum levels was moderate, the association is clinically relevant and points to the need for targeted interventions addressing both mobility limitations and vitamin D insufficiency among older adults [30].

Patterns of vitamin D supplementation among participants provided further insights into modifiable behaviors. Notably, 43% of individuals reported no use of vitamin D supplements. Among those who did supplement, the average daily intake was 505.99 ± 284.26 IU. Statistical analysis indicated a significant positive impact of supplementation on serum vitamin D status (*P* = 0.044) [31]. However, the variability in serum response suggests inconsistencies in absorption, bioavailability, or compliance, highlighting the need for individualized approaches to supplementation, particularly in the presence of comorbidities [32].

Calcium supplementation also demonstrated a significant, albeit smaller, effect on vitamin D levels (*P* = 0.039), potentially reflecting synergistic interactions between calcium and vitamin D in bone metabolism. In contrast, dietary patterns-including vegetarian, Westernized, traditional Pakistani, and mixed diets-did not significantly affect serum 25(OH)D concentrations (*P* = 0.71). This suggests that in this specific population, supplementation practices and existing health conditions may exert a more dominant influence on vitamin D status than dietary intake alone [33].

A notable finding was the association between a family history of osteoporosis and reduced serum vitamin D levels (*P* = 0.043). This may point to underlying genetic or behavioral factors that affect vitamin D metabolism or skeletal health across generations [34]. Conversely, the presence of prior non-hip fractures did not correlate significantly with current vitamin D levels (*P* = 0.86), possibly because such events occurred under different physiological conditions and may not reflect present-day nutritional status [35].

Surprisingly, neither hypertensive status nor history of smoking showed a significant relationship with serum vitamin D levels (*P* = 0.834). This outcome diverges from some studies that have reported an inverse association between smoking and vitamin D concentrations, potentially mediated through oxidative stress or hepatic metabolism [36]. The lack of significant associations in this study could stem from the presence of confounding variables that obscure these relationships [37].

Seasonal variability, often implicated in fluctuating vitamin D levels due to changing sunlight exposure, was not found to be significant in this cohort (*P* = 0.68). This may be indicative of a consistently indoor lifestyle that limits photoconversion of vitamin D year-round. These findings raise important questions regarding the adequacy of endogenous vitamin D synthesis during periods of low solar radiation, especially in populations that depend heavily on exogenous sources for their nutrient needs [38].

The study identified a range of comorbid conditions-including diabetes mellitus, CKD, thyroid disorders, and gout-as being significantly associated with serum vitamin D concentrations. Logistic regression analysis particularly highlighted diabetes and gout as major predictors of deficiency, in line with other studies that document complex interactions between chronic disease states and nutrient metabolism [39].

Biochemical analyses further revealed elevated levels of CRP (50.24 ± 16.36 mg/L), suggestive of systemic inflammation, which may negatively influence vitamin D metabolism [40]. Concurrently, higher serum uric acid and reduced hemoglobin levels were observed, indicative of metabolic burden and potential undernutrition [41]. These findings point to the importance of considering vitamin D status within a larger clinical framework that includes inflammatory and nutritional biomarkers [42,43].

Cluster analysis allowed for the stratification of participants into low-, moderate-, and high-risk groups based on clinical parameters. The high-risk subgroup was characterized by elevated CRP, urea, and creatinine levels, in conjunction with the lowest serum 25(OH)D values. This risk-based categorization supports the role of vitamin D not only as a nutritional marker but as a broader indicator of systemic health and resilience in the elderly [44].

Overall, this study highlights the multifactorial origins of vitamin D deficiency in older adults suffering hip fractures. Key modifiable factors, such as BMI, mobility, sunlight exposure, and supplement use, are critical in determining serum vitamin D status. Yet these do not operate in isolation. The intricate interplay between inflammation, renal function, comorbid conditions, and nutritional adequacy presents a complex picture of vitamin D metabolism and fracture vulnerability [45].

The study advocates for the conceptualization of vitamin D as both a modifiable risk factor and a predictive biomarker in strategies aimed at fracture prevention. Given the consistently low serum levels observed and the limited efficacy of standard supplementation protocols, there is an urgent need to revisit current dosage recommendations, particularly for individuals identified as high risk [46,47]. The observed clustering of vitamin D deficiency with renal impairment and inflammatory markers further supports the integration of vitamin D screening within broader geriatric assessment frameworks [48].

Ultimately, the findings underscore the necessity for comprehensive public health interventions that address vitamin D and calcium intake while also accounting for the broader determinants of health. These include comorbidity burden, mobility status, nutritional access, and lifestyle behaviors that collectively influence bone integrity and fracture risk in the aging population [49,50].

This study presents several limitations that should be considered when interpreting its findings. The cross-sectional design inherently restricts the ability to establish causal relationships between vitamin D levels and hip fracture risk, as it only provides a snapshot of data at a single point in time. Additionally, the use of a convenience sampling method at a single center (Mardan Medical Complex) may lead to selection bias, as the study participants may not fully represent the wider elderly population, especially those from different geographical locations or healthcare settings. The exclusion of participants with recent immunosuppressive therapy or high-dose vitamin D supplementation further limits the generalizability of the findings, as these individuals may have distinct vitamin D metabolism characteristics. The study also relies on self-reported data for dietary habits, lifestyle choices, and sun exposure, which are susceptible to recall bias and inaccuracies. Moreover, the serum vitamin D levels were assessed only once, within 72 hours of hospitalization, which may not accurately reflect the participants’ long-term vitamin D status, particularly since acute conditions could influence these levels. Variability in vitamin D supplementation doses and adherence could also introduce inconsistencies in results, making it difficult to draw definitive conclusions regarding the effectiveness of supplementation. While biochemical markers like CRP, urea, and creatinine were measured, other potential confounding factors, such as genetic influences, other medications, or comorbid conditions like hyperparathyroidism, were not fully accounted for. Furthermore, the population's homogeneity, in terms of ethnicity and geographic location, limits the ability to generalize the findings to more diverse populations with different environmental, lifestyle, or genetic factors.

## Conclusions

The study suggests that vitamin D deficiency is common within this population, with factors such as BMI, mobility status, and comorbidities potentially influencing bone health. However, the exact interplay between these elements remains unclear. While vitamin D supplementation is recognized for its role in bone health, its effectiveness appears to vary significantly among individuals, indicating that a one-size-fits-all approach may not be sufficient. Although factors like age and gender were not found to have a significant impact on vitamin D levels or fracture risk in this cohort, the study highlights the importance of public health interventions that focus on regular vitamin D screening, tailored supplementation, and addressing comorbidities. These findings point to the potential of vitamin D as a predictive marker for fracture risk and a modifiable risk factor, though further research is needed to better understand the underlying mechanisms and optimize preventive strategies for older adult patients.
